# Crossed Unfused Ectopic Pelvic Kidneys: A Case Illustration

**DOI:** 10.1155/2018/7436097

**Published:** 2018-05-07

**Authors:** Jad A. Degheili, Murad M. AbuSamra, Fadi El-Merhi, Albert El-Hajj

**Affiliations:** ^1^Division of Urology, Department of Surgery, American University of Beirut Medical Center, Riad El-Solh, Beirut 1107 2020, Lebanon; ^2^Department of Diagnostic Radiology, American University of Beirut Medical Center, Riad El-Solh, Beirut 1107 2020, Lebanon

## Abstract

Crossed unfused ectopia constitutes a very rare variant of ectopic kidneys, with an approximate incidence of 1 : 75000. We hereby describe a rare case of an incidental finding of crossed unfused ectopic kidneys, in a 45-year-old gentleman incidentally found to have a bladder lesion. The unique blood supply of his kidneys has also been described. The present case also highlights the different subtypes of renal ectopia, the different embryological hypotheses behind their presentation, and the various systematic anomalies, associated with them. Variations in vasculature of ectopic kidneys have been only described in case reports and are crucial to recognize in case any further intervention is needed.

## 1. Introduction

Pronephros, mesonephros, and metanephros represent the three primitive kidneys, present during the various developmental stages of the human embryo. These are, respectively, located from the cephalic to the caudad position [1]. During the fifth week of gestation, the metanephros, which will later be the definitive kidney, is formed. The excretory units composed of the glomeruli and the collecting system originate from the metanephric mesoderm and ureteric bud, respectively. Soon after during the sixth and seventh gestational week, the ureteric bud will then expand into a metanephric tissue and then dilate and split to form the renal pelvis and the major calyces [2].

During the growth of the embryo, the metanephric mesoderm, initially located near the cloaca, gradually ascends to a more cephalad position, in the retroperitoneal area; as such, the blood supply will directly be from the aorta, whereas the more caudal supplying branches vanish. Persistence of such caudal branches will lead to “accessary” branches that may supply the inferior or superior pole of the kidneys [1].

The umbilical arteries constitute the “arterial fork,” which may hinder the ascent of the metanephros kidneys; as such, they remain in the vicinity of the iliac vessels, referred to as ectopic pelvic kidneys. When such kidneys cross to the contralateral side, opposite the ureteral insertion into the bladder, and then fuse/do not fuse with the other kidney, then they are referred to as crossed fused/unfused kidneys; for this, multiple postulates are proposed [3].

We hereby report a rare case of ectopia manifested as crossed unfused kidneys, with their peculiar blood supply, followed by a description of the various hypotheses that stand behind this phenomenon. Associated systematic anomalies with renal ectopias are also mentioned.

## 2. Case Report

**Figure 1:**
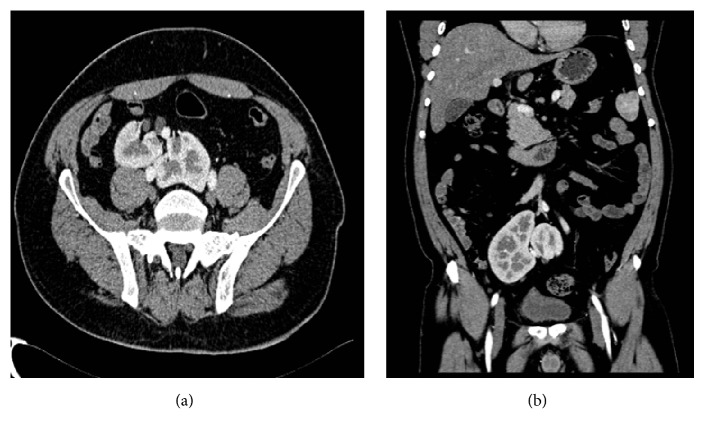
Axial (a) and coronal (b) CT scan, with intravenous contrast in the corticomedullary phase, showing a clear plane of separation between the two kidneys.

A 45-year-old gentleman, heavy smoker, presented with intermittent episodes of gross hematuria, over the past two weeks. Physical examination and laboratory tests were unremarkable. Enhanced computed tomography with delayed phases was requested, along with cystoscopy. The latter revealed a polypoid lesion, located within the right lateral wall, which was resected; CT-urography revealed the presence of ectopically located kidneys with no evidence of hydronephrosis, calculi, or parenchymal lesions. Both kidneys were located along the right paramedian area within the pelvis, with no evidence of fusion (Figure 1). The ureters appeared unremarkable with normal anatomical implantation within the bladder.

**Figure 2:**
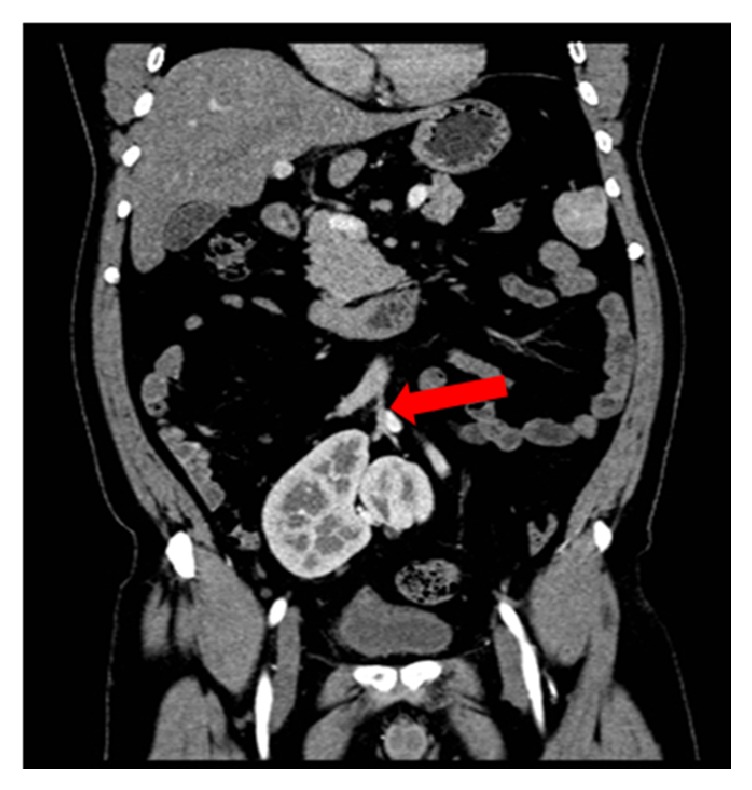
Coronal CT scan with IV contrast showing the median sacral artery (red arrow) giving right and left renal arteries.

**Figure 3:**
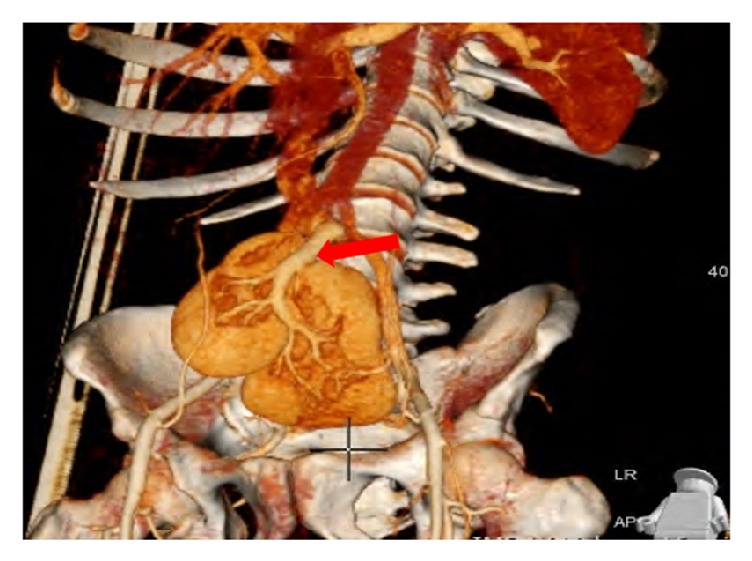
MDCT with VRT (volume rendering technique) showing the crossed unfused ectopic kidneys with the presence of joined draining vein (red arrow) of both kidneys.

Performing a 3D reconstruction, on the obtained images, for better visualization of the renal vasculature, the following was revealed. The right kidney had two arteries: the first originating from the proximal right common iliac artery and the second originating from the median sacral artery. On the other hand, there were two arteries supplying the left kidney, branching from the distal left common artery and the median sacral artery (Figure 2). Right and left renal veins joined to form one vein, which drained in the left common femoral vein (Figure 3).

## 3. Discussion

Crossed renal ectopia is a rare entity detected incidentally in 20 to 30% of cases [4] and results from the aberrant migration and crossing of the midline by the metanephric blastema and the ureteral bud [5], usually occurring during the fourth to eighth week of gestation [5]. Despite the ectopic position of the kidneys, the ureters are not crossed and insert normally into the bladder [4].

Renal ectopia is generally classified either as a simple renal ectopia or as a crossed renal ectopia (CRE) [6]. Second in incidence after horse-shoe kidney [5], with male-to-female predominance of 1.4 to 2 : 1, with two to three times higher chance to have a left-to-right ectopy [7], CRE is further categorized into four subcategories: crossed fused renal ectopia (CFRE), unfused ectopia, solitary CRE, and unfused bilateral CRE [4]. Among the four subcategories, those with fusion represent the majority of cases, around 90% [4], with an incidence of 1 in 2000 [6], followed by the unfused form with an incidence of 1 : 75000 [5]. There are 6 subtypes of CFRE: inferior, sigmoid or S-shaped, lump, disc, L-shaped, and superior CRFE [5]. The inferior form is the most common among the CFRE, and the superior form is the least common [5].

Several hypotheses have been used to shed light on the precise mechanism behind the development of renal fusion anomalies. Among those hypotheses, the mechanical theory, the genetic theory, the ureteral theory, and the theory of abnormal caudal rotation are described.

The “mechanical theory” describes the reason for renal ectopia as secondary to an altered configuration of the arterial fork. As such, this may render both kidneys in close proximity that may result in fusion, as is the case with horse-shoe kidneys [8]. This abnormal position of the umbilical arteries may result in migration of a kidney to the contralateral side, following the path of the least resistance. Eventually, a crossed ectopia is formed [8].

Since renal ectopic anomalies are observed in identical twins and siblings of the same family, then there should be an additional genetic basis for this phenomenon. Mutations in the sonic hedgehog (SHH) gene form the cornerstone behind the “genetic theory” of renal ectopia and fusion [3].

A wandering ureteral bud may cross the midline to the contralateral side and diffuse with the contralateral metanephric blastema, rather than the same side blastema, resulting in the formation of a crossed ectopic kidney. The other blastema, which did not fuse, will soon regress. Such hypothesis formed the basis of the “ureteral theory” of ectopia [8].

Last but not least, the “theory of abnormal caudal rotation” proposes an explanation for renal ectopia, by stating that the lateral flexion and rotation of the caudal end of the embryo will thus alter the position of the blastema, relative to the ureteric bud, rendering them crossed. Supporting this theory is the association of renal ectopia with spine scoliosis [8].

Most cases of ectopic kidneys are asymptomatic and detected incidentally, after investigation for other etiologies. Renal ectopia may be associated, with a relatively increased risk, with urinary tract infection, renal calculi, and ureteropelvic junction obstruction, which most likely is attributed to mechanical causes, malrotation, and aberrant vasculature [9]. Individuals with ectopic kidneys may witness a higher incidence of vesicoureteral reflux, which occurs in 20% of crossed kidneys. Other associated abnormalities include megaureters, duplication of collecting systems, hypospadia, cryptorchidism, posterior urethral valve, and cystic dysplasia with unilateral agenesis of fallopian tubes and ovaries [4]. Association with skeletal (radial club hand, hemivertebrae, spina bifida, scoliosis, and congenital hip dislocation), cardiopulmonary, and gastrointestinal anomalies (imperforate anus and esophageal atresia with tracheoesophageal fistula) [10], along with various genetic syndromes, has been also described [9].

Variation of vasculature, in ectopically located kidneys, has only been described in few case reports [11–14]. On the contrary to the normal embryological degeneration of the caudal vessels upon migration of the kidneys to their usual retroperitoneal location, renal vessels, in ectopic kidneys, do not degenerate; thus, several variations in blood supply may arise, resulting in more than one accessory and polar artery [1]. Knowledge of such variations is crucial prior to performing any surgical or radiological intervention.

## Abbreviations

CRE: Crossed renal ectopia
CFRE: Crossed fused renal ectopia.
